# Complications During Hospitalization in Patients With SARS-CoV-2 Pneumonia in a Romanian Pulmonary Center

**DOI:** 10.7759/cureus.33882

**Published:** 2023-01-17

**Authors:** Alexandra M Cristea, Dragos C Zaharia, Stefan Leu, Miron A Bogdan

**Affiliations:** 1 Pneumology, Marius Nasta Institute of Pneumology/ Carol Davila University of Medicine and Pharmacy, Bucharest, ROU; 2 Pneumology, Marius Nasta Institute of Pneumology, Bucharest, ROU; 3 Pneumology, Carol Davila University of Medicine and Pharmacy, Bucharest, ROU

**Keywords:** coronavirus pandemic, coronavirus disease, global pandemic, pandemic, complications, infection, covid-19, sars-cov-2 pneumonia

## Abstract

Introduction

The coronavirus disease (COVID-19) was declared a pandemic by the World Health Organization (WHO) on March 11, 2020. Facing a new and unknown virus, the entire medical community made considerable efforts to find a specific treatment, develop guidelines, and even create a vaccine. Besides all the measures taken, a wide range of complications associated with the disease increased the mortality and morbidity rates, adding more difficulty to the management of the patients.

Study design

We performed a retrospective study, including the patients with SARS-CoV-2 pneumonia who were admitted to our hospital between March 2020 and August 2021. We analyzed complications that developed during the hospitalization, such as respiratory failure or acute injury to other organs (the heart, pancreas, kidneys, and liver), and whether they were treatment- and hospitalization-related.

Results

One thousand eight hundred and forty-four cases were evaluated, and we analyzed the complications that developed during the hospitalization. Out of this, 1392 (75.48%) cases developed at least one complication during hospitalization, most frequently respiratory failure (71.14%), hyperglycemia (43.54%), renal injury (42.67%), or cardiovascular events (7.10%).

Conclusion

SARS-CoV-2 infection in hospitalized patients with pneumonia can cause injuries to any organ, making the management of those patients even more difficult.

## Introduction

The Severe Acute Respiratory Syndrome Coronavirus 2 (SARS-CoV-2) pandemic, declared by the World Health Organization (WHO) on March 11, 2020 [1], had devastating consequences both for the medical community and the entire population of the planet. When the first cases were discovered in December 2019 in Wuhan, China, they initially appeared to be caused by just another virus with respiratory tropism affecting a population that had already had contact with severe acute respiratory syndrome (SARS) and Middle East respiratory syndrome (MERS) in previous years. The unexpected surprise stemmed from its high contagiousness, rapid global spread, and the toll it has taken on healthcare systems around the world.

The global economy was greatly shaken by the measures that were implemented in an attempt to prevent the spread of the pandemic. Besides the economic perspective, people were also affected by the emotional impact of being separated from their loved ones and not being able to have physical interactions due to the quarantine imposed. Faced with a new and aggressive pathogen, the medical community from all over the world had to find prevention measures, possible therapeutic options, and vaccine development in a very short time, all while hospitals were overcrowded with an ever-growing number of severe patients, many of whom required ICU admission, while those with non-COVID conditions had to be postponed. Aside from that, many patients experienced a wide range of unpredictable and often difficult-to-treat complications during their hospitalization.

## Materials and methods

We performed a retrospective observational study and analyzed complications developed by patients with SARS-CoV-2 pneumonia during hospitalization in a mono-disciplinary pneumo physiology first-line COVID-19 hospital between March 2020 and August 2021, a period corresponding with the first three waves of the pandemic. All participants provided written informed consent prior to enrollment in the study. This study was approved by the Marius Nasta Institute of Pneumology Ethics Committee, Bucharest, Romania.

Inclusion criteria were: confirmed infection by either a specific viral antigen, a polymerase chain reaction (PCR) test, or an increased titer of specific antibodies - immunoglobulin G (IgG) or immunoglobulin M (IgM) - for SARS-CoV-2 S protein; ground glass consolidations suggesting viral pneumonia identified on chest radiography or chest computed tomography.

The patients’ discharge documents were analyzed, and if they met the inclusion criteria, data were entered and processed in Microsoft Office Excel, obtaining quantitative variables (numerical and percent).

## Results

Out of a total of 2837 patients that were hospitalized between March 2020 and August 2021, 1844 were included, recording clinical features and biological blood tests on admission, in-hospital treatment, comorbidities, the need for an intensive care unit, and complications developed during hospitalization. 760 were women, with a median age of 62.04 years (minimum age 18 and maximum age 99), and 1084 were men, with a median age of 59.43 years (minimum age 19 and maximum age 93). Smoking status is not mentioned.

Out of 1844 patients, 1392 (75.48%) presented with at least one complication while they were hospitalized.

Respiratory failure was diagnosed when either oxygen measured peripherally (with a pulse oximeter) dropped below 90% or when the patient required oxygen supplementation. A total of 1319 patients, representing 71.52% of the total number of cases, developed respiratory failure while they were infected with the SARS-CoV2 virus.

Biological parameters obtained by performing blood tests on admission were: blood glucose level, hepatic enzymes, and creatinine. Hyperglycemia was considered at a value >126 mg/dl, hepatic injury at twice the upper limit of the normal values (50 UI) of aspartate aminotransferase (AST) and alanine aminotransferase (ALT), and kidney injury were evaluated by the glomerular filtration rate (GFR) calculated with the Modification of Diet in Renal Disease (MDRD) formula. Table 1 presents the complications of the entire lot, either specified in the medical records or identified after data analysis, including the type of complication and the number of cases in which the specific complication was identified, both as an absolute number and as a percentage.

**Table 1:** Complications developed during hospitalization in patients with SARS-CoV-2 pneumonia PE- Pulmonary embolism; CDI: clostridium difficile infection; MODS- multiple organ dysfunction syndrome; ICU- intensive care unit

Complication type	Total number of complications - % of complications	Specific complication	Number of cases - % of cases	Comments
Respiratory	1352 - 73.31%	Respiratory failure	1319 - 71.52%	
		Pneumomediastinum	16 - 0.86%	
		Pneumothorax	12 - 0.65%	
		Other (alveolar hemorrage, pulmonary abcess, hypercapnia, pleuresy)	5 - 0.27%	
Metabolic	1392 - 75.48%	Hyperglycemia	803 - 43.54%	
		Hepatic	235 - 12.74%	
		Renal	787 - 42.67%	
Cardiovascular	131- 7.10%	Thromboembolic events	89 - 4.82%	Confirmed PE: 42 cases
				Suspicion of PE: 26 cases
				Stroke: five cases
				Acute limb ischemia: eight cases
				Coronary acute syndrome: five cases
		Arrhythmias	26 - 1.4%	Venous thrombosis: three cases
		Others	16 - 0.86	Hypo/hypertension, pectoral angina
Gastrointestinal	44 - 2.38%	Enterocolitis	36 - 1.95%	Diarrhea: 28 cases; CDI suspicion: eight cases
		Others	8 - 0.43%	Acute pancreatitis, fulminant acute liver failure, nausea, vomiting, pain
Neurological	28 - 1.51%	Psychomotor agitation	13	
		Neuropathy	6	
		Others (insomnia, disorientation)	9	
Nosocomial infections	37- 2%	CDI	13	
		Pulmonary	12	
		Urinary	5	
		Others	7	
Bleeding	34 - 1.84%	Gastrointestinal	12	
		Epistaxis	10	
		Pulmonary	8	
		Others	4	
MODS	35 - 1.89%		35	34 cases in the ICU
Other complications	49 - 2.65%	Acid-base disorders	19	Nine cases of metabolic acidosis
		Others	30	

The changes we observed in the biochemical samples obtained from blood tests performed at admission are presented in detail in Table 2. Hyperglycemia was identified in 803 cases (43.54%), hepatic injury in 235 cases (12.74%), and kidney injury in 787 cases (42.67%).

**Table 2:** Detailed biochemical modifications in patients with SARS-CoV-2 pneumonia ALT: alanine aminotransferase; AST: aspartate aminotransferase; GFR: glomerular filtration rate

Organ affected	Biochemical parameter	Number of cases	Details about a previously known disease or treatment	
			Findings	Number of cases
Pancreas	Glycemia	803	Received corticotherapy during this hospitalization	770
			Diabetes mellitus	315
			Treatment with insulin	87
			Oral antidiabetic agents	191
Liver	Transaminases ALT and AST	235	viral infection	Seven (five with the B virus, two the with C virus)
			mixt cirrhosis	Six
			others (dislipidemia, hydatid cyst removed, treatment toxicity)	Five
Kidney	GFR (ml/min/1.73m^2^)	787	CKD without hemodialysis	32
			CKD with hemodialysis	10
			Others (chronic infections, nephrectomy, renal cancer, lithiasis)	31

## Discussion

Respiratory failure was the most frequent complication in our lot. It can be the consequence of many pathophysiological modifications: injuries of the vascular endothelium, micro-thrombosis, the pulmonary coexistence of both shunt and dead space ventilation in the case of pneumonia [2], or a massive pulmonary embolism. This may explain why patients respond extremely differently to the administration of supplemental oxygen, with some having a favorable outcome with only a small amount of oxygen while others develop acute respiratory distress syndrome (ARDS), requiring high-flow oxygen therapy, mechanical ventilation, and even extracorporeal membrane oxygenation (ECMO). Out of a total of 1379 patients with respiratory failure, 756 had previously known cardiovascular diseases, with chronic heart failure (CHF) being identified in 72 of them. Five of the patients with CHF had a diagnosis of chronic respiratory failure, and three of them also had stage IV COPD. Regarding chronic pulmonary disease, 196 patients had either a history of a pulmonary condition or chronic lung disease, out of which 63 had COPD and 14 also had chronic respiratory failure. Another three patients were known to have chronic respiratory failure caused by other comorbidities. All the patients with chronic oxygen therapy had an increase in the amount of oxygen needed to maintain the saturation above 90%, and we considered the aggravation of hypoxia caused by viral infection.

Another respiratory complication is the presence of air in the mediastinum (pneumo-mediastinum) or pleural space (pneumothorax). In our study, the incidence of pneumothorax was 0.65% (16 cases) of the total number of cases, which is similar to the one found in a study that included 3368 patients with COVID-19 disease and pneumonia. The incidence was 0.66%, and no connection between mechanical ventilation and the presence of pneumothorax was established [3]. There was also no link found between mechanical ventilation and pneumomediastinum; however, the presence of air in the mediastinal space is a sign of a poor prognosis. The pathophysiological mechanism may be explained by the effort produced by the cough, which can lead to ruptures of the tracheal wall or the breaking of the alveoli as a consequence of pneumonia. The incidence we found in our study was 0.86%, which is comparable to another study including 58484 patients, where an incidence of 0.64% was found [4].

Regarding the management of these complications, several actions have been taken: transitioning from non-invasive ventilation to oxygen delivered by a facial mask as soon as possible; lowering ventilator pressures or oxygen flow as low as the patient’s condition allows; and achieving a normal oxygenation level (target SpO2 >92%). All cases of pneumothorax were treated surgically by inserting a drain tube into the chest wall.

As presented in Table 2, biological changes on admission were found in a similar percentage (75.48%). Hyperglycemia was considered when the value of blood sugar à jeun was higher than 126 mg/dl, which already satisfies diagnostic criteria for diabetes mellitus, according to the American Diabetes Association [5]. While half of the patients (803) presented with hyperglycemia on admission, only 315 of them had been previously diagnosed with diabetes mellitus. Some of the mechanisms involved may be: first-time diagnosis; pancreatic injury caused by a viral infection; or cortisone treatment, given the fact that 770 out of 803 patients received corticosteroids, as required by national-approved guidelines. It is important to mention that blood tests were performed at admission and theoretically before any treatment was given, but in some cases, we were unable to perform laboratory tests during the weekend, and the gravity of the case imposed the beginning of the corticoid treatment. Involvement of the pancreas in the context of SARS-CoV2 infection was analyzed in a study that included 10 patients with COVID-19 who had developed hyperglycemia and revealed postmortem lesions of the pancreas [6].

Kidney injury associated with SARS-CoV2 infection leads to a longer hospitalization period, increased severity of the disease, and a poor prognosis [7]. We analyzed GFR in patients using MDRD-4=175 × (Scr)-1.154 × (Age)-0.203 × (0.742 if female) × (1.178 if black) [8], where Scr represents serum creatinine. We classified chronic kidney disease as normal or high >= 90 ml/min/1.73 m2, mildly decreased 60-89 ml/min/1.73 m2, mildly to moderately decreased 45-59 ml/min/1.73 m2, moderately to severely decreased 30-44 ml/min/1.73 m2, severely decreased 15-29 ml/min/1.73 m2, and kidney failure<15 ml/min/1.73 m2 [9]. Our findings are presented in Table 3.

**Table 3:** Kidney injury according to CKD classification by GFR GFR- glomerular filtration rate; CKD- chronic kidney disease

GFR (ml/min/1.73 m^2^)	Number of cases	Previously known CKD
60-89	520	16
45-59	139	12
30-44	79	12
15-29	30	7
<15	19	15

To avoid overestimation of kidney function or underestimation of kidney injury, we only looked at patients with creatinine levels above the hospital's upper normal limit [10]. In our study, only 62 (7.87%) of the 787 patients with an abnormal GFR had previously known chronic kidney disease, which supports the theory that the kidney injury was mostly due to viral infection. Our findings contradict a hypothesis that renal complications are predominant in cases of pre-existing chronic kidney disease [11]. A future approach to evaluating the renal function in a SARS-CoV2 patient may provide more accurate information by using more variables such as body mass index (BMI) or urinary output.

Hepatic injury was present in 235 cases (12.74%), defined as an increase above two times the upper limit of transaminase AST and ALT laboratory values, which can also be increased in cases of heart injury [12]. Injury of the liver may also be the consequence of inflammation caused by the viral infection or thrombosis; however, the specific mechanism is difficult to properly identify in the absence of specific tests. Anemia, hypoxia, or a decrease in blood flow to the liver can all have an effect on transaminase levels [13]. The severity of the disease is correlated with a higher level of hepatic enzymes [14], with an AST value two to three times higher than normal [15]. There is no established threshold for a link between hepatic injury and the value of enzymes, and in some studies, it was defined as any value higher than the normal laboratory upper limit [16], while other studies set a threefold increase above the upper limit value [17]. In our study, only 18 (7.65%) had previously known hepatic disease, most frequently caused by a viral infection or ethanol abuse. This may suggest that the hepatic injury was caused by the viral infection.

Cardiovascular complications were also significant. As a consequence of a strong inflammatory response to infection, cytokines are released into the bloodstream, causing injury to the endothelium and also to myocardial cells [18]. This cytokine storm causes embolism, myocarditis, or acute coronary syndromes due to plaque rupture or the release of excessive catecholamines. The toxicity of some of the treatments used for SARS-CoV-2 infection may also cause heart injury, especially with the intense use of corticosteroids [19]. In our study, the majority of cardiovascular complications were thromboembolic events and arrhythmias. Forty-two cases (2.27%) suffered a pulmonary embolism, and eight cases suffered acute limb ischemia, for which the recommended treatment was amputation. In our study, out of 26 patients who presented arrhythmias, atrial fibrillation was identified in 17 of them, which was also the most frequent arrhythmia reported in the literature in patients with SARS-CoV-2 infection [20].

Clostridioides difficile infection (CDI) is one of the most feared nosocomial infections, and patients with COVID-19 disease are at a high risk of developing it during hospitalization [21]. Diarrhea is also one of the main symptoms of SARS-CoV-2 infection, due to the fact that the digestive tract contains a large number of receptors to which the virus can bind [22]. Dysbiosis, one of the conditions causing diarrhea, can be developed by patients during hospitalization, either as a consequence of the treatment received or secondary to nosocomial infection. While in our study the main gastrointestinal complication was enterocolitis (1.95%), we only found a percentage of 0.7% of CDI, which we consider to be an underestimate of the real number of cases, perhaps caused by an insufficient number of stool tests performed.

It is also important to mention that the nosocomial infections encountered in our study are not directly related to viral infections. CDI cases were most likely a consequence of antibiotic treatment, while nosocomial infections involving the respiratory tract were recorded in patients admitted to the intensive care unit.

The risk of bleeding is increased in hospitalized patients by multiple factors, such as anticoagulant treatment or vessel injury caused by inflammation [23]. In our study, we found three organs mostly affected by this complication: the gastrointestinal tract, nose, and lungs. Gastrointestinal bleeding is most frequently a consequence of gastric and duodenal ulcers [24], while epistaxis is more likely caused by the administration of high-flow oxygen. A study conducted on patients receiving oxygen through a high-flow nasal cannula (HFNC) discovered an incidence of 10% for epistaxis [25]. Hemoptysis is most likely secondary to a pulmonary embolism. Because anticoagulant treatment is the most likely cause of bleeding, it is critical to carefully consider the risks and benefits of administration and dosage for each patient.

Acid-base disorders were reported in 19 cases (1.03%), nine of them being metabolic acidosis. Both respiratory and kidney failure lead to blood pH disruptions, leading to respiratory and, respectively, metabolic alkalosis [26]. Along with hyperglycemia, these pH-modifying conditions were frequently identified in our study and are an argument that acid-base disorders were probably underreported in our lot of patients.

Other complications were: urological (acute urine retention, dysuria); dermatological (urticaria, erythema, papules); surgical (acute abdomen); treatment-related toxicity: three cases linked to lopinavir/ritonavir (nausea, vomiting) and one linked to hydroxychloroquine (prolonged QT segment on electrocardiogram); three cases of intravascular disseminated coagulation; and four cases of post-viral complications: autoimmune hemolytic anemia, suspicion of encephalitis, pericarditis, and arthritis.

Our study presents some limitations. One of them is a lack of information about the timing of corticosteroid treatment initiation, given that many people self-medicate cases where we were unable to perform blood tests over the weekend and the gravity of the case imposed the start of corticoid treatment. All of these circumstances make establishing a link between hyperglycemia and virus-caused pancreatic injury difficult. A1c glycated hemoglobin determination would also be useful to determine if there was unknown diabetes, but this test was unavailable in our clinic. Another limitation is represented by the impossibility to evaluate the renal function of the patients in the absence of weight, body mass index, or 24- or 48-hour urinary output.

## Conclusions

The SARS-CoV-2 virus affects the respiratory system, leading to pneumonia complicated by respiratory failure. However, there are many other complications that may occur, including pneumomediastinum or pneumothorax, bacterial infection, or pancreatic injury. During hospitalization, these patients can develop injuries in any organ: the heart, blood vessels, liver, and kidneys, while the most severe cases suffer from the feared multiple organ dysfunction syndrome, which frequently leads to death. Besides those directly related to the virus infection, the management of a hospitalized patient with SARS-CoV-2 pneumonia has many other challenges, such as nosocomial infections, toxicity, or adverse reactions to the specific treatment.
